# Modulation of Neuroinflammation by the Gut Microbiota in Prion and Prion-Like Diseases

**DOI:** 10.3390/pathogens10070887

**Published:** 2021-07-13

**Authors:** Josephine Trichka, Wen-Quan Zou

**Affiliations:** Department of Pathology, Case Western Reserve University, 2103 Cornell Rd, Cleveland, OH 44106, USA; jjt78@case.edu

**Keywords:** neuroinflammation, microbiota, dysbiosis, innate immunity, prion disease, Parkinson’s disease, Alzheimer’s disease, prion-like disease

## Abstract

The process of neuroinflammation contributes to the pathogenic mechanism of many neurodegenerative diseases. The deleterious attributes of neuroinflammation involve aberrant and uncontrolled activation of glia, which can result in damage to proximal brain parenchyma. Failure to distinguish self from non-self, as well as leukocyte reaction to aggregation and accumulation of proteins in the CNS, are the primary mechanisms by which neuroinflammation is initiated. While processes local to the CNS may instigate neurodegenerative disease, the existence or dysregulation of systemic homeostasis can also serve to improve or worsen CNS pathologies, respectively. One fundamental component of systemic homeostasis is the gut microbiota, which communicates with the CNS via microbial metabolite production, the peripheral nervous system, and regulation of tryptophan metabolism. Over the past 10–15 years, research focused on the microbiota–gut–brain axis has culminated in the discovery that dysbiosis, or an imbalance between commensal and pathogenic gut bacteria, can promote CNS pathologies. Conversely, a properly regulated and well-balanced microbiome supports CNS homeostasis and reduces the incidence and extent of pathogenic neuroinflammation. This review will discuss the role of the gut microbiota in exacerbating or alleviating neuroinflammation in neurodegenerative diseases, and potential microbiota-based therapeutic approaches to reduce pathology in diseased states.

## 1. Introduction

Over the past decade or so, dysregulation of the gut microbiota (dysbiosis) has been shown to have widespread effects on systemic homeostasis and response to disease. Advances in next-generation sequencing have allowed for large-scale correlational studies wherein specific microbiota profiles have been linked to susceptibility to infectious diseases, certain autoimmune conditions such as inflammatory bowel disease, malignancy, and blood–brain barrier (BBB) dysfunction [1]. Alternatively, promotion of some commensal bacterial populations can have beneficial systemic effects such as neuroprotection after stroke [2], enhancement of metabolism [3], and production of vitamins critical for DNA synthesis and blood coagulation. Fecal microbiota transplantation, wherein stool from a healthy donor is transplanted into the colon of the recipient, is becoming a common therapeutic approach to managing conditions such as *Clostridium difficile* infections [4] and inflammatory bowel disease [5] that have a microbiota-associated etiology. These concepts have been recently applied to the study of neurodegenerative diseases, where it has been determined that dysbiosis in the gut can result in enhancement of neuroinflammation in the CNS, while gut microbiota homeostasis promotes a steady state in the CNS.

## 2. Gut Microbiota and Neuroinflammation

Neuroinflammation is defined as “activation of neuroimmune cells into proinflammatory states,” [6] and has been associated with the pathogenesis of almost every neurodegenerative disease. It can occur in both the brain and spinal cord, and in many cases, it is unclear whether a given neurodegenerative disease precipitates neuroinflammation and/or whether neuroinflammation is a key player in the pathogenesis of that disease. The two primary cell types that mediate neuroinflammation are microglia and astrocytes, which produce cytokines, chemokines and reactive oxygen species that cause local inflammation and recruit peripheral immune cells across the BBB. Microglia are essentially the resident macrophages of the brain, and perform homeostatic functions such as synaptic pruning as well as immune surveillance and phagocytosis. Microglia, unlike peripheral blood monocytes, have extended processes that permit them to continuously survey their microenvironment and establish contact with neuronal synapses [7]. Upon Toll-like receptor (TLR) activation by a pathogen-associated molecular pattern (PAMP) or damage-associated molecular pattern (DAMP), microglia undergo a process called microgliosis, where their cytoplasmic processes swell to give the cell a more amoeboid appearance [8]. While this phenotypic alteration and the associated release of cytokines and pro-inflammatory factors is beneficial in the context of a short-term threat to the central nervous system (CNS), neurodegenerative diseases that produce constant triggers can cause unchecked microglial activation that results in widespread damage to the CNS. Under homeostatic conditions, astrocytes carry out multiple beneficial functions within the CNS including provision of growth factors to neurons, regulation of synapse formation and plasticity, and regulation of the composition of extracellular fluid; in addition to maintenance of the BBB [9]. Under pathologic conditions, they are involved in activating the adaptive immune response via release of cytokines and chemokines. Interestingly, studies have indicated that the commensal gut microbiota can regulate functional maturation of microglia and microglial function is compromised in germ-free mice that lack the commensal gut microbiota [10].

The etiologies of neuroinflammation are diverse, and include infection, traumatic brain injury (TBI), accumulation of toxic metabolites, autoimmunity, and proteopathic seed aggregation. In the case of local ischemia caused by microvascular disease or TBI, ATP release from damaged or dying cells is sensed by purinergic receptors on microglia, resulting in microglial activation [11,12]. Infection and pathogenic protein aggregation, on the other hand, trigger microglial activation via release or presentation of PAMPs (infection) and DAMPs (protein aggregates) that trigger microglial TLRs. Activated microglia release reactive oxygen species (ROS), reactive nitrogen species (RNS), glutamate, locally active cytokines, and peripherally active chemokines [11,12]. ROS, RNS, and glutamate are neurotoxic in large quantities and therefore lead to local tissue damage. They also, alongside pro-inflammatory cytokines, result in the propagation of inflammation within the CNS by activating neighboring microglia and astrocytes. These pro-inflammatory mediators are also responsible for altering the BBB permeability such that chemokines and inflammatory chemoattractants can recruit additional peripheral immune cells to the CNS. The key commonality to the onset of chronic neuroinflammation is a chronic exposure to an inflammatory stimulus that results in dysregulation of innate immunity in the CNS. The inflammatory response, initially intended to limit the spread of infection and reduce the threat posed by cellular debris and/or misfolded protein aggregates, is usually restrained by tolerogenic elements that oppose inflammation and restore homeostasis. In the context of a chronic inflammatory stimulus, however, such as prolonged exposure to pathogenic protein aggregates, this tolerogenic response is suppressed and neuroinflammation can propagate indefinitely.

## 3. Gut Microbiota and Blood–Brain Barrier

The BBB is designed to protect the CNS from toxins, immune cells, and pathogens that could damage its parenchyma and disrupt its functionality, while allowing the passage of nutrients into and waste out of the CNS. In the case of systemic inflammation, however, the permeability of the BBB becomes altered, and this can result in the accumulation of neurotoxic waste and an increased influx of peripheral immune cells into the CNS. Although they are disparate sites, disruption to the composition of the gut microbiota can result in systemic inflammation capable of disrupting the integrity of the BBB or exacerbate existing inflammation in the CNS that has already resulted in altered BBB permeability. Under homeostatic conditions, however, the presence of a pathogen-free gut microbiome can actually promote the maintenance of an intact BBB. A 2014 study found that germ-free mice had increased BBB permeability compared to pathogen-free mice with normal gut flora, and that exposing these germ-free mice to a normal gut microbiota could restore BBB impermeability [13]. The state of BBB permeability is determined largely by the expression of transmembrane tight junction proteins such as occludin, claudin-5, and JAMs by specialized endothelial cells. These tight junction proteins maintain an intact barrier by forming tight junction strands that create intercellular contacts by interacting with cytoplasmic scaffolding proteins [14]. An additionally important factor in BBB maintenance is the regulation of these tight junction proteins by pericytes, which are contractile cells embedded in the vascular basement membrane of the endothelium.

## 4. Gut–Brain Axis

There are numerous conditions that can result in dysbiosis, many of which involve intestinal pathologies, but some of which do not. Intestinal disorders that can cause dysbiosis include inflammatory bowel disease (Crohn’s disease and ulcerative colitis), irritable bowel syndrome (IBS), and celiac disease. Colonization of the gut by inflammatory bacteria such as *Heliobacter pylori* is an additional established origin of dysbiosis, and such colonization has been known to cause systemic inflammation that can exacerbate many pathologies, including neurodegenerative diseases such as Parkinson’s disease (PD) and Alzheimer’s disease (AD) (which are the subjects of this review) [15,16,17]. Extra-intestinal dysbiosis-causing disorders include allergy, asthma, metabolic syndrome, and cardiovascular disease [18,19] While there is a good deal of variability between organisms in terms of their microbiome composition, there remains a “core microbiome” in every individual that comprises approximately 40% of all microbial genes present. The phyla that constitute this core microbiome are *Bacteroides* and *Firmicutes*, which are the dominant phyla, in addition to *Proteobacteria*, *Actinobacteria*, *Fusobacteria*, *Spirochaetes*, *Verrucomicrobia*, and *Lentispherae* [20].

The manner in which this core microbiome communicates with the CNS in the steady-state largely involves the production of small molecules and metabolites by commensal bacteria that promote CNS homeostasis. For example, microbiome-derived short-chain fatty acids (SCFAs) regulate microglial homeostasis and also induce regulatory T cells (T_regs_) to combat CNS autoimmunity [1,10]. It has also been reported that these SCFAs improve memory and enhance synaptic plasticity via inhibition of histone deacetylase [21]. Other bacterial metabolites that contribute to CNS homeostasis include branched-chain amino acids (BCAAs), which have roles in neurotransmitter synthesis and regulation of food intake [22], and peptidoglycans, the presence of which suppresses inflammatory responses to commensal bacteria and modifies the secretion profile of TLR-expressing cells in the CNS [23]. Tryptophan metabolism also figures prominently into microbiome-CNS communication, as products of tryptophan metabolism by gut bacteria such as indole, tryptamine, kynurenine, quinolinate, indole acetic acid, and indole propionic acid are neuro-active and capable of modulating CNS activity [24].

Additional players at the microbiome-CNS interface include the enteric nervous system and the vagus nerve, which provide a direct connection between the gut and the CNS. The commensal microbiome is critical for proper development of the enteric nervous system, and thereafter, the enteric nervous system communicates with the CNS via the vagus nerve. The vagus nerve is capable of sensing microbial metabolites via its afferent components within the enteric nervous system, and then transmitting this information to the CNS for integration into the central autonomic network [25]. In this manner, information concerning alterations in microbiota composition is quickly transmitted to the CNS, where the appropriate response can be initiated. As an example of this line of communication, one hypothesis concerning the pathogenesis of PD involves the retrograde transport of α-synuclein from the enteric nervous system through the vagus nerve to the dorsal motor nucleus of the vagus as a result of pathogen-induced α-synuclein misfolding in the gut epithelium [26]. Gut dysbiosis could therefore directly contribute to PD pathogenesis by causing the production in the gut and subsequent transmission of a known pathogenic protein into the CNS.

## 5. Parkinson’s Disease

PD, the second most common neurodegenerative disease after AD, is characterized by loss of neuronal mass from the substantia nigra and α-synuclein aggregates located within intracellular inclusions, which form Lewy bodies. These pathological injuries result in dopaminergic deficiency, which in turn causes the predominant clinical findings of bradykinesia, rigidity, tremor, and postural instability [27]. The most common form of PD is idiopathic due to the lack of known causative genetic mutations; thus, the elucidation of environmental triggers is of high importance to the understanding of this disease [28]. Interestingly, gastrointestinal manifestations such as constipation and gastroparesis are also associated with non-motor prodromal signs and symptoms of PD, providing one of the original rationales for looking into the relationship between the microbiota and the pathogenesis of PD. A recently published meta-analysis that summarized the findings of 10 independently published studies on differential microbiota composition between PD patients and healthy control subjects showed that different bacterial phyla predominate in the PD patients’ microbiome [29]. Specifically, the increased presence of rarer bacterial species causes a decrease in the abundance of species that more commonly populate the gut, and this replacement predisposes PD patients to lower levels of gut butyrate (caused by reduced Roseburia, Fusicatenibacter, Blautia, Anaerostipes, and Faecalibacterium species), increased gut inflammation and permeability (caused by increased Akkermansia species), and increased methane production (caused by increased Methanobrevibacter species) [29]. An additional recent study correlates the abundance of opportunistic pathogens in the gut with mutations in the SNCA gene, which encodes for α-synuclein, showing that host genotype can influence gut seeding by different microbial populations [30].

Beyond these human studies showing a correlation between microbiota composition and the onset of PD, there are also murine studies in a PD model that manipulate the microbiome to investigate its relationship to the pathogenesis of PD. Alpha-synuclein overexpressing (ASO) mice with a complex microbiota, compared to germ-free ASO mice, displayed impaired gross motor function, fine motor control, and striatal function [31]. The germ-free mice also displayed decreased α-synuclein aggregates compared to mice with a complex microbiota. One explanation for this relationship between the microbiota and PD clinical findings is the increased prevalence of gram-negative lipopolysaccharide (LPS)-producing bacteria in the microbiota of PD patients. LPS is a potent trigger of inflammation, and acts by activating TLR4 on innate immune cells. The prevalence of gram-negative bacteria such as Enterobacteriaceae has been shown to correlate with the degree of postural instability and gait difficulty in PD patients [32]. Gram-negative bacteria are known to produce significant quantities of LPS, and LPS has been shown to produce PD-like pathology when injected into the substantia nigra [33]. Based on this observation, studies have investigated the relationship between enteric LPS production and PD pathology and shown that intraperitoneal administration [34], oral administration, and intrarectal administration [35] of bacterially derived LPS can produce PD pathology. LPS administration has also been demonstrated to correlate with decreased ZO-1 and e-Cadherin expression and increased intestinal permeability in an ASO mouse model [36]. Besides LPS production, SCFA production by gut microbes has also been linked to PD pathogenesis. Oral SCFA administration in ASO mice was sufficient to cause increased microglial activation and increased motor deficits compared to an untreated control group [31]. These data contrast the long-held hypothesis that SCFAs are protective in neurodegenerative disease [37,38] suggesting that perhaps SCFAs can play different roles in different pathological contexts.

While it has been shown that intestinal inflammation and resulting release of neuroactive substances from the gut can affect PD pathology, it remains unclear whether these substances act via communication with the peripheral nervous system or traffic to the CNS to interact directly with the microglia that are ultimately activated by their production. SCFAs are capable of crossing the BBB via monocarboxylate transporters on endothelial cells [39]; however, peripherally derived LPS cannot cross the BBB in quantities sufficient to explain its neuroactive properties and ability to excite microglia in a wild-type mouse [40]. It has been proposed that toxins and metabolites secreted by gut microbes could travel in a retrograde manner through the vagus nerve to the brain, and that these harmful compounds could specifically alter the mitochondrial integrity of CNS neurons [41]. DAMPs released by mitochondria could then propagate neuroinflammation by activating Toll-like receptors (TLRs) on neighboring microglia. Interestingly, α-synuclein has been shown to accumulate in the enteric nervous system prior to the development of motor symptoms in PD patients [42], and has subsequently been shown in mice to be capable of retrograde propagation from the gut to the brain via the vagus nerve in a prion-like manner [43]. A recent study provides evidence that a cell surface amyloid protein called curli that is produced by gut-resident Escherichia coli is capable of accelerating α-synuclein aggregation in the gut and brain and promoting both GI dysfunction and motor impairment as a result [44]. Once in the brain, oligomeric α-synuclein contributes to PD pathogenesis through its canonical mechanism: toxic protofibrils cause disruption of cellular homeostasis and induction of neuronal death through inhibition of synaptic signaling, alteration of cytoskeletal dynamics, loss of protein degradative ability, and mitochondrial fragmentation [45]. This propagation of α-synuclein from the gut to the brain was originally proposed to be the cause of idiopathic PD in 2003 [46]. However, it has also been proposed that there exist two different etiologies of PD: a brain first “top-down” etiology and an enteric nervous system first “bottom-up” etiology. The group that proposed this hypothesis was able to generate supporting clinical data in PD patients, showing that the trajectory of the disease could be divided into two subtypes based on the pattern of loss of cholinergic innervation, cardiac sympathetic innervation, integrity of pigmented locus coeruleus neurons, and putaminal dopamine storage capacity [47]. 

It has been suggested that gastrointestinal biopsies could serve as biomarkers for early stages of PD based on detection of α-synuclein aggregates [48]. However, even with early detection, clinical interventions that would halt the progression of PD remain out of reach. Current therapies for PD are focused on exogenous replacement of dopamine and inhibition of dopamine degradation pathways. If we accept the existence of both a top-down and a bottom-up etiology of PD, it is possible that manipulation of the microbiota in bottom-up early-stage PD could alleviate neuropathology. In support of this therapeutic approach, two separate retrospective cohort studies followed patients with truncal vagotomies and found an almost 50% reduction in risk of developing PD [49,50]. With more and more studies investigating the species that are either beneficial or harmful to PD patients, probiotics may eventually come to play a more prominent role in the treatment of PD patients. Clinical trials suggest that cocktails of bacteria tailored to replace the species most commonly lost in PD may yield a favorable response, although these results must still be confirmed by additional studies [51]. Another approach to altering the gut microbiota in PD patients is via fecal transplantation, an approach that has yet to be explored in humans but has shown to be beneficial in a murine model [52]. Perhaps the simplest method of promoting a healthy microbiota and preventing exacerbation of PD pathogenesis, though, is via manipulation of dietary factors. In humans, a Mediterranean-type diet with emphasis on consumption of fruits, vegetables, and whole grains was shown to lower risk of PD diagnosis [53]. A comprehensive review performed in 2019 compiled data suggesting that in addition to a Mediterranean diet, neuroprotection could be conferred by uric acid and poly-unsaturated fatty acids, while milk increases the risk of developing PD [54].

## 6. Alzheimer’s Disease

The primary disease-causing mechanism in AD is the accumulation of a pathogenic Aβ peptide variant capable of forming plaques that are proposed to lead to deposition of tau, synaptic loss, neuronal death, and cognitive decline [55]. While AD can only definitively be diagnosed by examination of brain tissues post-mortem, the clinical presentation associated with its diagnosis is characterized by progressive episodic memory loss, executive dysfunction, language deficits, and neuropsychiatric changes [56]. Biomarkers are sometimes also used in diagnosis, with Aβ_42_/Aβ_40_ and total/phosphorylated tau used most commonly [56]. Neuroinflammation plays a central role in AD pathology: inflammation self-propagates due to interactions between neurons, microglia, astrocytes, Aβ plaques, and hyperphosphorylated tau-containing neurofibrillary tangles [57]. There are few studies that investigate the composition of the microbiota in AD patients, and little is known about the bacterial species that exacerbate or alleviate AD pathology. A 2018 study, one of the few performed, found that Bacteroides species were of lower abundance in AD patients, while Ruminococcaceae species were increased [58]. However, a previous study found increases in Bacteroides as well as decreases in Firmicutes and Bifidobacterium in the guts of AD patients compared to a control group without AD [59]. This study also observed correlations between commonly used AD CSF biomarkers such as Aβ_42_/Aβ_40_, phosphorylated tau, and chitinase-3-like protein and relative abundance of bacterial populations that differed from control microbiota composition. Future studies should investigate the compounds and toxins that these abnormal bacterial populations produce, their effects on AD pathogenesis, and their potential route of access to the CNS. 

Murine models of AD have recapitulated the observation that microbiota composition differs between AD and non-AD individuals [60,61,62,63], an important step in ensuring that findings from these mouse studies will translate to a greater understanding of the microbiota in the context of human AD. Based on the proposed role of the microbiota in the pathogenesis of AD, studies in mouse models of AD have manipulated the microbiome to determine the outcome on AD CNS pathology. Germ-free AD mice were found to have less severe AD pathology than mice with conventional microbiota, measured by decreased levels of Aβ peptide deposition, decreased microglial activation, and increased levels of Aβ-degrading enzymes [63]. Perhaps the most convincing evidence of the microbiota’s influence on AD pathology from this study is an experiment showing that restoration of germ-free mice with a complex, AD mouse-derived microbiome increases pathological findings of AD [63]. Antibiotic treatment, too, has been shown to decrease amyloidosis and microglial activation in a murine model of AD [64], further confirming the importance of the microbiome in AD pathogenesis. 

The microbes that comprise the human microbiome produce large amounts of LPS and functional amyloid, which are thought to play a role in the pathogenesis of AD [65,66]. For example, the curli fibers produced by E. coli that were found to accelerate α-synuclein aggregation are a class of functional amyloid proteins that are involved in bacterial biofilm formation and gut colonization [67]. In an environment replete with toxic, proinflammatory amyloid deposits, microglia become overwhelmed and cannot effectively clear debris. This leads to immune activation, chronic inflammation, and oxidative stress that all contributes to the pathogenesis of AD. Studies have now shown that in addition to direct Aβ_42_ contributions to neuroinflammation, bacterial LPS and endotoxins can also exacerbate the neurotoxicity in AD by promoting amyloid fibrillogenesis [68,69]. Interestingly, the functional amyloid produced by microbes is recognized by TLR2 expressed on microglia, which is the same TLR that recognizes Aβ_42_ [70]. Additionally, CD14 expressed on microglia recognizes both microbial PAMPs and Aβ_42,_ highlighting the role of the innate immune response in the pathogenesis of AD [71]. It has been suggested based on these observations that molecular mimicry could play a role in the activation of the innate immune system: Aβ_42_ may resemble bacterial PAMPs and/or secreted compounds to the extent that it causes aberrant activation of microglia in the CNS [66]. While there is no direct evidence to support the assertion that microbial amyloid may traffic from the gut to the CNS, researchers have used the confirmed retrograde transport of α-synuclein through the vagus nerve as proof of concept of this phenomenon [72].

Murine fecal transplant has showed promising results as a potential avenue of treatment for human AD, although clinical trials in humans have not yet been performed. Transfer and/or transplantation of fecal matter from non-AD mice into AD mice decreased Aβ plaque and neurofibrillary tangle formation, decreased neuroinflammation, and improved cognitive metrics [60,73]. These studies build on previous findings that the removal of a purportedly pathogenic microbiome from AD mice can ameliorate disease progression [63], by showing that replacement of the pathogenic microbiota with the microbiota from healthy donor mice can further reduce disease progression in a manner that possesses greater clinical relevance. Last year, an 82-year-old patient with AD who underwent fecal microbiota transplantation to resolve a Clostridioides dificile infection was evaluated after transplantation and found an improved mini-mental status exam score, as well as self-reported improvements in mood and memory [74]. While findings from one patient should not initiate a paradigm shift in our approach to treating AD, this case study should prompt further investigation of the efficacy of fecal matter transplantation as a treatment for AD on a much larger scale. As in PD, it has been suggested that dietary alterations could be beneficial in decreasing risk and improving the prognosis of AD. Pomegranate, for example, was demonstrated to have neuroprotective effects that are attributed to its containing urolithins, which have been shown to prevent Aβ fibrillation in vitro [75]. A study that used principal component analysis to assess the correlation between nutrient patterns consumed by patients and risk of developing AD found that diets with greater proportions of fruit, vegetables, whole grains, fish, and low-fat dairy were associated with lower risk than diets comprised of more sweets, fried foods, high-fat dairy products, processed meat, and butter [76], findings that were corroborated by an additional study [77]. The diet recommended by these studies closely resembles the Mediterranean diet thought to be neuroprotective against the development of PD. Notably, the Mediterranean diet is particularly high in phenol content, and multiple researchers tout the benefits of phenols in diet for AD risk management [78,79,80] based on in vitro studies demonstrating that phenols are capable of discouraging amyloid fibrillation [81,82]. Although the benefits of phenols have not been proven in clinical trials, the argument can be made that there is no harm in altering one’s diet in a manner that has been suggested by experts to be neuroprotective (and better for human health on the whole).

## 7. Prion Diseases

Compared to PD and AD, little is known about the role of gut microbiota in prion diseases. The effects of gut microbiota on prion diseases may be more complicated than on other neurodegenerative diseases. Like PD and AD, prion diseases also belong to the group of neurodegenerative disorders that are associated with the deposition of misfolded cellular proteins in the brain. However, different from PD that is associated with misfolded α-synuclein and AD that is associated with both misfolded amyloid β and tau, prion diseases are associated with an infectious scrapie prion protein (PrP^Sc^), formed from a cellular prion protein called PrP^C^ through a structural transition. The deposition of protease-resistant PrP^Sc^ in the brain first causes astrocyte and microglia activation, followed by spongiform degeneration and neuronal loss [83]. Moreover, prion diseases are transmissible and affect not only humans but also animals [84]. Human prion diseases can be spontaneous and have both sporadic and genetic forms, including sporadic Creutzfeldt-Jakob disease (sCJD), variably protease-sensitive prionopathy (VPSPr) and sporadic fatal insomnia as well as genetic CJD, Gerstmann-Sträussler-Scheinker (GSS) disease and fatal familial insomnia (FFI). They can also be acquired via infection with exogenous prions such as kuru and variant CJD through the gastrointestinal system as well as iatrogenic CJD via medical and surgical contamination [85]. Animal prion diseases include scrapie in sheep and goats, bovine spongiform encephalopathy or mad cow disease in cattle, and chronic wasting disease in deer and elk. It has been well-documented that mad cow disease is zoonotic. It has been transmitted to humans to cause variant CJD by the consumption of contaminated beef products from cattle with mad cow disease through the gastrointestinal route [86,87].

It is known that a large number of microbiota hosted in the mammalian intestine can have both beneficial and detrimental effects on host health. For instance, microbiota can participate in the metabolism of nutrients and the regulation of host development and can also safeguard against pathogens [88]. They are also believed to regulate the functional maturation of microglia through the gut–brain axis. Indeed, microglial function has been observed to be compromised when the commensal microbiota is absent in germ-free mice [10]. On the other hand, the gut microbiota-brain microglial interaction may trigger detrimental effects by facilitating prion disease pathogenesis [88,89,90].

In an early study, germ-free mice inoculated intracerebrally with the Chandler mouse-adapted prion isolate were found to exhibit a longer survival time compared to infected conventional control mice [89]. This result was believed to be due to an impaired status of microglia in germ-free mice [10] that impeded CNS prion pathogenesis [88]. Although a subsequent study by Wade et al. (1986) revealed no difference in survival time between germ-free and controls mice inoculated intracerebrally with ME7 scrapie prions, a longer survival time was observed in germ-free but not in control mice after intraperitoneal inoculation [90]. In contrast, Bradford et al. recently demonstrated that the absence of the commensal microbiota in germ-free mice did not affect prion disease duration or susceptibility after intraperitoneal or intracerebral injection of mouse-passaged 22C scrapie prions [91]. In addition, this study also found that the magnitude and distribution of the prion-characteristic neuropathological changes including spongiform degeneration and accumulation of PrP^Sc^ as well as astrogliosis and microglial activation in the brain exhibited no differences between conventional and germ-free mice. It was concluded that dramatic changes to the abundance or complexity of the commensal gut microbiota are unlikely to influence prion disease pathogenesis [91]. The reasons behind the inconsistencies among above studies remain unclear.

There are even fewer studies to investigate the effect of prion diseases on the gut microbiota. It has been noticed that patients with prion disease often have gastrointestinal disorders including loss of appetite and constipation, and dehydration [88]. Interestingly, a new study by Yang et al. suggested that prion disease may affect the gut microbiota. Compared to healthy controls, the gut microbiota in fecal samples of infected mice revealed increased numbers of Proteobacteria and less Saccharibacteria at the phylum level and more Lactobacillaceae and Helicobacteraceae and less Prevotellaceae and Ruminococcaceae at the family level [92]. One hundred and forty-five fecal metabolites were significantly different in mice with prion infection, 114 of which were lipid metabolites. They also found that three phosphatidylcholine compounds dramatically decreased and four hydrophobic bile acids considerably increased. Decreases in eight types of short-chain acids and increases in Cys and Tyr and decreases in His, Trp, and Arg were observed in mice with prion infection [90]. These findings suggest that prion disease may cause shifts in the gut microbiota. It would be interesting for future studies to determine whether and how these changes in the gut microbiota will affect the pathogenesis of prion diseases.

## 8. Discussion

**Figure 1:**
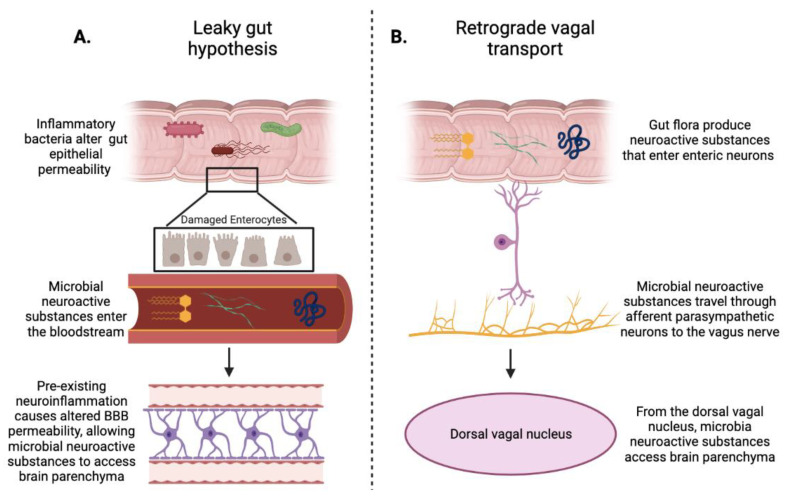
Potential avenues through which neuroactive microbial products could reach the CNS. (**A**) the leaky gut hypothesis necessitates the pre-existence of both gut and neurological inflammation. Inflammatory microbes in the gut cause local inflammation, which damages enterocytes such that integrity of the endothelial barrier is diminished. Gut microbes produce endotoxins and neuroactive substances, including neurotransmitters, that permeate the damaged gut epithelium and enter the bloodstream. Neuroinflammation in the CNS, usually caused by ongoing innate immune reaction to abnormal protein accumulation and aggregation, causes loss of blood–brain barrier integrity. Microbial substances can thus enter the CNS and exacerbate neuroinflammation. (**B**) Gut microbial substances enter afferent enteric neurons and are transmitted via the vagus nerve to the dorsal vagal nucleus in the CNS. Created with BioRender.com (accessed on 12 July 2021).

There is definitive evidence that the murine microbiota is capable of modulating the pathogenesis and clinical severity of PD and AD, and additional correlational evidence in humans demonstrating that patients with PD or AD have a different gut microbiota composition from healthy control study participants. There seem to be two likely mechanisms at play in the propagation of neurodegenerative disease by the gut microbiota: firstly, the induction of systemic inflammation by a pro-inflammatory gut environment and the allowance of this by increased permeability of the gut epithelium (the well-known “leaky gut” hypothesis; Figure 1A) [93]. This mode of transmission also requires alteration of the BBB to admit microbial endotoxins and amyloid proteins into the CNS, however, which usually does not occur in the steady state. For this propagation to occur, then, neuroinflammation must be pre-existing and have disturbed the integrity of the BBB. While it is tempting to propose a “chicken or egg” approach to the understanding of the etiology of these prion-like diseases, it seems most likely that these processes occur independently of one another and subsequently work together to worsen disease. The second likely candidate mechanism to explain the link between the microbiota and CNS pathology is the physical connection between the enteric nervous system and the CNS: the vagus nerve (Figure 1B). In this theory (which is not independent of the first theory introduced), inflammatory mediators such as α-synuclein produced by gut bacteria travel in a retrograde manner from enteric neurons through the vagus nerve to the dorsal nucleus of the vagus. This mechanism is more compatible with a “gut-first” spread of neurodegenerative disease, but as Horsager et al. note in their study, patients seem to show signs of multiple etiologies leading to the onset of neurodegenerative disease [47].

**Figure 2:**
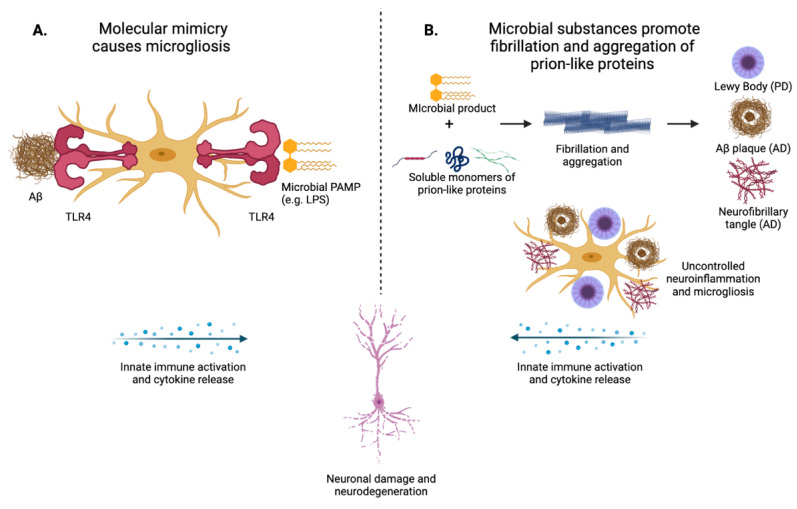
Innate immune activation resulting from communication along the gut–brain axis. (**A**) Microbial PAMPs and Aβ share a domain that binds and activates TLR4, leading to the theory that molecular mimicry could be promoting innate immune recognition of Aβ and subsequent propagation of neuroinflammation. Neuroinflammation increases Aβ production and aggregation, causing a vicious cycle known as the amyloid cascade hypothesis. (**B**) Microbial products like endotoxin and functional amyloid promote fibrillation and aggregation of prion-like proteins such as α-synuclein, tau, and Aβ. These prion-like proteins, once aggregated, form insoluble aggregates (amyloid plaques, neurofibrillary tau tangles, and Lewy bodies) that overwhelm microglial phagocytic capacity, thereby promoting aberrant immune activation and neuroinflammation. Created with BioRender.com (accessed on 12 July 2021).

In both proposed etiologies of prion-like diseases, however, there is sparse evidence to support the localization of bacterial-derived mediators within the CNS in disease-burdened patients, although murine and in vitro studies demonstrate worsening pathologies when these substances are introduced directly into a neural context. A 2020 study showed that circulating levels of LPS in the blood correlate to worse amyloid pathology in AD [94], but as of yet there is insufficient evidence that these molecules actually accumulate in the brains of patients with neurodegenerative diseases. In murine models as well, most studies show either that increased production of microbial products correlates to worse CNS pathology or that facilitation of the interaction of these products with neurons and/or microglia worsens pathology. Proposed models explaining the instigation of neuroinflammation by interaction between secreted microbial products and the CNS microenvironment include molecular mimicry (Figure 2A) and the promotion of fibrillation of prion-like proteins by secreted microbial products (Figure 2B). These models both necessitate the physical presence of microbial endotoxins and secreted microbial products within the CNS. Future research should attempt to parse out the contributions of systemic microbiota-mediated inflammation versus direct effects of microbial products that have circumnavigated the BBB and entered the CNS on the pathology of neurodegenerative diseases.

Cohort studies have shown that diet can prove beneficial in ameliorating symptoms and/or risk of both PD and AD, and there are multiple ongoing clinical trials to investigate the use of probiotics in decreasing disease acquisition risk, but clinical manipulation of the microbiota via fecal matter transplantation (FMT) has not yet been thoroughly studied. As of May 2021, according to clinicaltrials.gov, there is only one clinical trial of FMT for the treatment of PD, for which results have not been published yet, with three studies recruiting or in progress. For FMT in AD, there are no completed studies and only one study in progress. FMT is a relatively safe procedure, and it is possible to cure almost all complications of FMT with antibiotic therapy alone [95]. A recently published systematic review found that of 4241 patients who underwent FMT, the most common complications were diarrhea and abdominal discomfort with the incidence of both being less than 10% [96]. Furthermore, the incidence of adverse events was only 1.4%, with five (0.1%) of those adverse events resulting in death from mucosal barrier disruption. It seems prudent to engineer more clinical trials focused on the use of FMT to treat PD and AD based on the definitive participatory role of the microbiome in the pathogenesis of these two diseases.

In conclusion, although novel research concretely implicates dysbiosis in the pathogenesis of the prion-like Parkinson’s disease and Alzheimer’s disease, the mechanism by which inflammation propagates from the gut to the CNS is unclear, as is the potential for manipulation of the gut microbiota to serve as a clinical therapy for the treatment of these diseases. As is becoming the case in many diseases, a holistic view of systemic homeostasis is providing critical insights into disease pathogenesis and potential avenues for treatment. These systemic interactions must be thoroughly investigated and taken advantage of from a treatment perspective to serve the betterment of human health.
